# Evaluation of single and multiple hyaluronic acid injections at different concentrations with high molecular weight in the treatment of knee osteoarthritis

**DOI:** 10.1186/s12891-024-07200-y

**Published:** 2024-02-21

**Authors:** Selim Safali, Erkan Sabri Ertaş, Ali Özdemir, Deniz Cataklı

**Affiliations:** 1 Orthopaedics and Traumatology Department, Faculty of Medicine, Selcuk University, Konya, Turkey; 2 Department of Pharmacology, Faculty of Medicine, Suleyman Demirel University, Isparta, Turkey

**Keywords:** Intra-articular injections, Hyaluronic acid, Knee osteoarthritis

## Abstract

**Background:**

Knee osteoarthritis is severe progressive and most commonly diagnosed articular disease and its incidence is increasing around the world depending on age. This pathologic condition which limits daily activity of patients can be characterized by degeneration of cartilage and inflammation. Although non-steroidal anti-inflammatory (NSAII) agents and other analgesics are routinely used treatment options, the potential effects of intraarticular injections including hyaluronic acid (HA) have also been demonstrated by various studies. However, few studies compare the efficacy of a single high molecular weight (HMW) high dose and a triple HMW low dose. This study aimed to compare the efficacy of single high molecular weight (HMW) high dose (2 mL / 60 mg) and triple HMW low dose (2 mL /30 mg) intra-articular injection of HA in knee osteoarthritis (OA) patients by evaluating function and pain parameters during 12 months.

**Methods:**

This is a single-center, retrospective clinical study that included and involved 128 patients. Group I (*n*=64) patients received triple 30 mg HA injections (SEMICAL®) with one-week intervals, while Group II (*n*=64) patients received a single 60 mg HA injection (SEMICAL®). Lequesne Index, WOMAC and VAS scores were recorded to assess pain and function during a 12-month period.

**Results:**

There was no significant difference in characteristics of patient demographics. Our finding indicate that WOMAC, VAS score, and Lequesne Index values during follow-up visits exhibited a decrease, signifying improvement in the clinical condition. Notably, scores were significantly more favorable with the 30 mg of HA injection compared to the 60 mg of HA injection.

**Conclusion:**

This study suggests that the triple low-dose injection of HMW HA is more effective in improving WOMAC, VAS scores and Lequesne Index values than a single high-dose injection.

## Background

Knee osteoarthritis (OA) is a severe progressive and most frequently diagnosed articular disease that affects 250 million people around the world [1]. In the rank of global disabilities, knee and hip OA place at the 11th and the incidence of OA increases in older ages [2, 3]. This pathologic condition is characterized by degeneration of cartilage and inflammation, and has symptoms that limit daily activity and life quality such as pain, stiffness and functional deterioration [4]. Also, it affects healthcare costs considerably.

There are widely used treatment options for OA including non-steroidal anti-inflammatory drugs (NSAID), other analgesics, intra-articular corticosteroids, local therapies, hyaluronic acid (HA) and surgery as well [5–7]. Various physical agent modalities are also utilized in the treatment of OA with a low level of evidence, but their effects are limited [8]. New investigations are ongoing, such as intra-articular Botulinum toxin injection, which has shown promising results in the early stages, aiming to manage pain in OA [9], Over time, intraarticular injections that include corticosteroids, ozone, HA, and platelet-rich plasma (PRP) [10–14] have started to be preferred instead of NSAID due to less systemic side effects and faster pain relief effects [15]. The intra-articular HA injection, which is a commonly utilized treatment approach, has been demonstrated to be effective and has been shown to reduce the necessity for analgesic use [16].

HA is a polysaccharide that is involved in the cartilage and synovial fluid with various properties such as protection of cartilage, lubrication, and shock absorption of the joint [17]. In OA patients, inflammation leads to depolymerization and clearance of high rates of endogenous synovial HA [18]. Furthermore, molecular weight, concentration, source of origin of HA, dosage, cross-linkage and formulations can lead to variation in the biological activities of HA [19]. Recent randomized clinical trials have demonstrated that an exogenous form of HA (Viscosupplementation) has beneficial effects in the treatment of knee OA to relieve symptoms [17, 20].

Intra-articular HA) preparations vary in molecular weight, with low molecular weight preparations (0.25-1 million Dalton) achieving a higher concentration in the joint and potentially reducing inflammation, but with lower elastoviscosity than native HA [21]. High molecular weight preparations (2-7 million Dalton) can improve fluid retention in the joint and possibly provide stronger anti-inflammatory effects [22]. The efficacy of HA treatment may depend on the preparation's rheological properties and molecular weight [23], with recent studies on the use of HA with different molecular weights for knee OA treatment yielding conflicting results but possibly favoring high molecular weight HA [24, 25].

However, although many studies have reported the effects of single HA injection, there are limited studies that compare single and multiple injections of HA in OA patients. In addition, most published clinical studies evaluating the efficacy of HA injections have observation periods in the 6-month range [26]. To the best of our knowledge in the literature, no study with high density and high HA content and a clinical follow-up of at least 12 months has been found.

In this retrospective study, we compared the efficacy of single high molecular weight (HMW) high dose (2 mL / 60 mg) and triple HMW low dose (2 mL /30 mg) intra-articular injection of HA in knee OA patients by evaluating the function and pain parameters during 12 months.

## Methods

### Study design

This study is a retrospective analysis of prospectively collected data. The study was conducted by the principles of the Helsinki Declaration and received approval from the local (2022/46). Before any study procedure, each patient provided written informed consent to participate. The study was conducted by a physician from March 2021 to March 2022. Before any study procedure, each patient provided written informed consent to participate.

### Inclusion and exclusion criteria

Patients diagnosed with grade 2 or grade 3 knee osteoarthritis (OA) according to the Kellgren-Lawrence grading system, who were admitted to our hospital aged between 50 and 60 years with knee OA symptoms for 12-18 months, were included in the study. Patients with the following conditions were excluded from the study: grade 1 and grade 4 knee osteoarthritis, neoplasia, cognitive impairment, history of any knee surgery, body mass index (BMI) greater than 30 and implantation in the same limb.

**Fig. 1:**
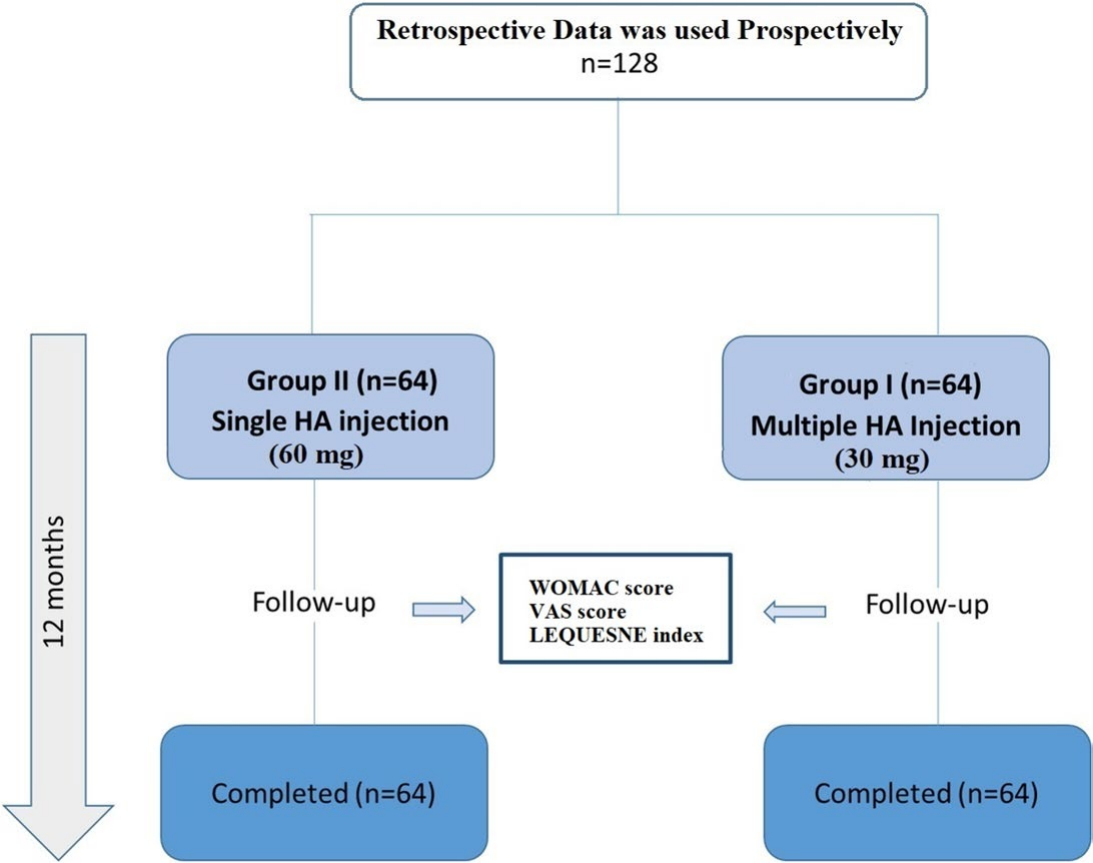
Study diagram

In this study, retrospectively collected patient information was used prospectively. Patients who applied to the clinic between March 1, 2021, and March 1, 2022, and received either a single dose of 60 mg HA or three doses of 30 mg HA were included in the study. In our clinic, routine intra-articular injections of either three doses of 30 mg HA or a single dose of 60 mg HA are administered. The treatment choice is adjusted based on the patient's preference. Two equal groups were formed by selecting patients with similar ages and weights from the group of patients who received injections and met our criteria. This was done to make the groups more similar and to obtain more reliable results. Subsequently, the results of these patients were examined in detail, and comparisons were made. A total of 128 patients, were included in the study. In group I, 64 patients were administered 3 doses of 30 mg of HA (SEMICAL^®^ HMW, linear unmodified HA, average molecular weight (Mw) 2.1 MDa Semikal Technology Incorporated Company) with-one week interval each .In group II, 64 patients received a single intra-articular knee injection of 60 mg of HA (SEMICAL^®^, HMW, linear unmodified HA, average molecular weight (Mw) 2.1 MDa Semikal Technology Incorporated Company).The study diagram is shown in Fig. 1.

### Interventions and clinical assessments

All injections were administered by the same surgeon. Patients who participated in the study were instructed not to take any anti-inflammatory drugs for 2-3 weeks before the first injection. Throughout these 2 weeks, patients followed standard exercise protocols, and no additional medication was administered. Before the injection, patients were seated with their knees relaxed and flexed at a 90-degree angle. Sterile gloves were worn, and the injection area was disinfected with povidone-iodine before the injection. Intra-articular injections were performed using twenty-two gauge (22G) needles.

All clinical evaluations were performed by a specialist who was blinded to the treatment group assignments. Clinical parameters including Western Ontario and McMaster Universities Arthritis Index (WOMAC) [27], visual analogue scale (VAS), [28] and Lequesne Index [29] were recorded to evaluate patients’ function and pain during follow-up visits at 1 month, 3 months, 6 months and 12 months after the last intra-articular injection.

### Statistical analysis

For statistical analysis the SPSS-22.00 package program was used. Shapiro-Wilk’s test was used to evaluate the normality of data. The chi-square test was applied to analyze the differences of these qualitative parameters. The paired t-test and independent t-test were used to compare mean values within and between the two groups, respectively. Differences were considered significant for *p*<0.05.

## Results

**Fig. 2:**
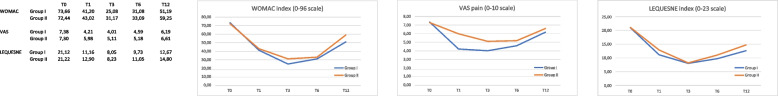
WOMAC, VAS, LEQUESNE index results were shown in graphics

A total of 128 patients with knee OA were included in the study, with 64 patients in group I receiving a triple 30 mg HA injection, and 64 patients in group II receiving a single 60 mg HA injection. Of the patients in Group I, 31 were male and 33 were female, with 43 having grade II knee OA and 21 having grade III knee OA according to the Kellgren-Lawrence classification. The mean age of patients in Group I was 58 years (range: 51-60), and in Group II, it was 57.9 years (range: 50-60). In Group II, there were 33 male and 31 female patients, with 41 individuals having grade II knee OA and 23 having grade III knee OA, as per the Kellgren-Lawrence classification. The mean BMI of patients in Group I 26.1 (range 23-30) , and in Group II , it was 25.8 (range 24-30). There were no significant differences in the demographic characteristics of the patients. The findings demonstrated a significant difference in WOMAC values between the follow-up visit intervals, with group I showing better values than group II at 1st, 3rd, 6th, and 12th months (Table 1). Moreover, VAS score values were significantly better in group I compared to group II at 1st, 3rd, 6th, and 12th months (Table 2). Similarly, Lequesne Index values were significantly better in group I compared to group II at 1st, 6th, and 12th months. According to the Lequesne Index values, no significant difference was found between Group I and Group II, at the 3rd month (Table 3). Additionally, all the results are presented with graphics (Fig. 2).

**Table 1:** Comparison of WOMAC scores between the groups based on treatment and follow-up visits. WOMAC index (0-96 point): The minimum overall WOMAC Index score is 0, indicating no symptoms or limitations, and the maximum score is 96 (20 for pain + 8 for stiffness + 68 for physical function), indicating the most severe symptoms and limitations across all subscales

**Time**	**Treatment**	**n**	$\overline{\mathbf{X} }$	**sd**	**sem**	**df**	**t**	***p***
**Before**	30 mg x 3	64	73.66	2.17	.271	126	3.28	.001*
	60 mg	64	72.44	2.04	.255			
**1**^**th**^** month**	30 mg x 3	64	41.20	1.89	.236	126	-4.74	.000*
	60 mg	64	43.02	2.41	.301			
**3**^**rd**^** month**	30 mg x 3	64	25.08	2.75	.344	126	-14.5	.000*
	60 mg	64	31.17	1.92	.239			
**6**^**th**^** month**	30 mg x 3	64	31.08	1.89	.237	126	-5.61	.000*
	60 mg	64	33.09	2.16	.270			
**12**^**th**^** month**	30 mg x 3	64	51.19	2.19	.274	126	-17.1	.000*
	60 mg	64	59.25	3.06	.383			

*Before the treatment’ (t= 3.28; p= .0001), “1*^*th*^* month” (t= -4.74; p= .000), “3*^*rd*^* month” (t= -14.5; p= .000), “6*^*th*^* month” (t= -5.61; p= .000) and “12*^*th*^* month” (t= -17.1; p= .000).* Cohen's d was calculated for the effect size estimation of the independent variables in the WOMAC. According to the Cohen's d results, the effect size was found to be 0.58 for the pre-treatment, 0.84 for the 1st month, 2.56 for the 3rd month, 0.99 for the 6th month, and 3.029 for the 12th month. Based on these values, it can be observed that the effect size was moderate for the pre-treatment and high for the other treatment time points

^*^p<0.05

**Table 2:** Comparison of VAS scores between the groups based on treatment and follow-up visits. VAS walking scale: (0-100 point) 0: no pain or normal walking, 100: worst possible pain or unable to walk

**Time**	**Treatment**	**n**	$\overline{\mathbf{X} }$	**sd**	**sem**	**df**	**t**	***p***
**Before**	30 mg x 3	64	73.8	2.1	.27	126	1.849	.067
	60 mg	64	73.0	2.2	.27			
**1**^**th**^** month**	30 mg x 3	64	42.1	2.2	.28	126	-47.97	.000*
	60 mg	64	59.8	1.7	.25			
**3**^**rd**^** month**	30 mg x 3	64	40.1	1.6	.19	126	-31.10	.000*
	60 mg	64	51.1	2.4	.30			
**6**^**th**^** month**	30 mg x 3	64	45.9	2.7	.33	126	-14.99	.000*
	60 mg	64	51.8	1.7	.21			
**12**^**th**^** month**	30 mg x 3	64	61.9	2.7	.34	126	-9.38	.000*
	60 mg	64	66.1	2.3	.29			

*‘Before the treatment’ (t= 1.849; p= .067), “1*^*th*^* month” (t= -47.97; p= .000), “3*^*rd*^* month” (t= -31.10; p= .000), “6*^*th*^* month” (t= -14.99; p= .000) and “12*^*th*^* month” (t= -9.38; p= .000).*Cohen's d was calculated for the effect size estimation of the independent variables in the VAS. According to the Cohen's d results, the effect size was found to be 0.37 for the pre-treatment, 9 for the 1st month, 5.39 for the 3rd month, 2.61 for the 6th month, and 1.67 for the 12th month. Based on these values, it can be observed that the effect size was moderate for the pre-treatment and high for the other treatment time points

^*^p<0.05

**Table 3:** Comparison of Lequesne Index between the groups based on treatment and follow-up visits. LEQUESNE index (0-24 point), 0: no pain or functional limitations, 24: severe pain and greater functional limitations

**Time**	**Treatment**	**n**	$\overline{\mathbf{X} }$	**sd**	**sem**	**df**	**t**	***p***
**Before**	30 mg x 3	64	21.12	1.96	.244	126	3.28	.001*
	60 mg	64	21.22	2.58	.323			
**1**^**th**^** month**	30 mg x 3	64	11.16	2.04	.255	126	-4.74	.000*
	60 mg	64	12.90	2.29	.286			
**3**^**rd**^** month**	30 mg x 3	64	8.05	1.60	.200	126	-14.5	.000*
	60 mg	64	8.23	1.88	.235			
**6**^**th**^** month**	30 mg x 3	64	9.73	2.06	.258	126	-5.61	.000*
	60 mg	64	11.05	2.07	.258			
**12**^**th**^** month**	30 mg x 3	64	12.67	2.61	.327	126	-17.1	.000*
	60 mg	64	14.80	2.64	.330			

*‘Before treatment’ (t= -.231; p= .817), “1*^*th*^* month” (t= -4.57; p= .000), “3*^*rd*^* month” da (t= -.607; p= .545) “6*^*th*^* month” (t= -3.59; p= .000) and “12*^*th*^* month” (t= -4.58; p= .000).*Cohen's d was calculated for the effect size estimation of the independent variables in the LEQUESNE index. According to the Cohen's d results, the effect size was found to be 0.04 for the pre-treatment, 0.80 for the 1st month, 0.1 for the 3rd month, 0.64 for the 6th month, and 0.81 for the 12th month. Based on these values, it can be observed that the effect size was moderate for the pre-treatment and high for the other treatment time points

^*^p<0.05

## Discussion

In this study, we aimed to compare the efficacy of a single high-dose high molecular weight (HMW) HA intra-articular injection with a triple HMW low-dose HA injection in knee osteoarthritis (OA) patients, with a follow-up period of 12 months. Through our investigation, we sought to obtain valuable insights into the most effective dosing strategy for HA injections in knee OA. Our results indicate that triple low-dose HA injections were more effective in reducing pain and improving function compared to single high-dose HA injections, as evidenced by the significant differences in WOMAC and VAS scores between the two groups. In the Lequesne Index, patients in Group I showed significant improvement compared to Group II, except in the 3rd month. In the treatment comparison, there is some advantage in favour of the multi-injections regime, without reaching a statiscally level (*p* > 0.05) in most of the cases. In our analysis, only the VAS shows a better result at short time (mainly at 1 month) but a risk of bias exists, as the first injections were done 2 weeks in advance, with the group I. This slight difference in favor of multi-injections could result from a total dose of HA injected : 90 mg vs 60 mg.

Our results support the superiority of a triple low-dose HA intra-articular injection at two-week intervals compared to a single high-dose HA injection. This finding may have significant implications for clinical practice. One notable consequence of a single high-dose HA injection is the potential reduction in the number of recurrent outpatient clinic visits. An additional advantage is the reduction in the number of injections for the patient, leading to a potential decrease in complications associated with the minimally invasive procedure. This would undoubtedly alleviate the medical workload and offer economic benefits. Moreover, it would benefit patients who are averse to frequent doctor visits. Due to psychological reasons, some patients might still prefer repeated injections despite the potential advantages of a single high-dose treatment. Our study findings also demonstrate that triple low-dose injection therapy is more effective.

In recent years, several studies have compared the effectiveness of different molecular weights of HA. Wu's meta-analysis, which summarized relevant studies, concluded that high molecular weight (HMW) HA injections demonstrated the best efficacy for up to 6 months after treatment without an increased risk of adverse effects [30]. Similarly, Concoff's meta-analysis found that intra-articular injections of HA used in a 2-4 injection treatment regimen provided the greatest benefit compared to IA-Saline with respect to pain improvement in patients with knee OA [31]. These findings align with our study, indicating the effectiveness of HMW-HA with both single and multiple injections. However, it's worth noting a study conducted by Carrabba et al. in 1992 [32], which evaluated the effectiveness of single-injection formulations of HA compared to 3- or 5-injection series in patients with knee osteoarthritis. The study reported a statistically significant improvement in the ISOAK score at 60 days favoring the 5 injections versus the single injection, but there was no difference between 3 injections and 5 injections. It is important to consider that the study had several limitations, including a relatively small sample size, inclusion of patients with effusion, loss of 80% of patients to follow-up after 4 months, and lack of volume adjustment.

In a prospective multicenter, randomized trial conducted by Conrozier et al. in 2009 [33], the effectiveness of variable volumes of Hylan G-F 20 (Synvisc) was evaluated over a 6-month period in 120 patients with unilateral knee osteoarthritis. The patients were divided into five groups, including a single 6-mL injection group and a single 4-mL injection group, as well as groups that received a combination of volumes given either 2 or 3 weeks apart. The study's findings indicated that the single 6-mL injection was equally efficacious and well-tolerated as 2 mL weekly for 3 consecutive weeks. Additionally, the 6-mL single-injection group had the lowest number of patients requiring retreatment. Although this study had a relatively small sample size and was not double-blinded, it utilized an appropriate FDA-approved volume for the single-injection formulation and demonstrated similar outcomes in terms of pain relief and functional improvement when compared with the multiple injection series. The study conducted by Al-Omran and Azam [34] aimed to compare the efficacy of three different treatments for knee osteoarthritis (OA): hylan G-F 20 (Synvisc, 3 injections), HA 60 mg/3 ml (Durolane, single injection), and HA 40 mg/2 mL (Osteonil, 3 or 5 injections). The study was a prospective, double-blinded, randomized trial involving 227 patients. The primary outcome measure was the change in WOMAC scores at 6 months. The results of the study demonstrated that hylan G-F 20 (Synvisc) was the most effective treatment among the three, showing a statistically significant improvement in WOMAC scores compared to both HA 40 mg/2 mL (Osteonil) and HA 60 mg/3 mL (Durolane). Additionally, the study found that HA 40 mg/2 mL (Osteonil) was also superior to HA 60 mg/3 mL (Durolane).

Controlling pain in knee osteoarthritis is associated with significant functional gains and improved quality of life for patients. Scaturro et al. [35] conducted a prospective study involving 37 patients with symptomatic knee osteoarthritis and a body mass index greater than 25. They evaluated the patients three months after ultrasound-guided intra-articular injection of hybrid HA complexes (Sinovial® H-L). The study found a statistically significant improvement in VAS, WOMAC score, and cardiopulmonary capacity after treatment. Additionally, there was a significant improvement in the patients' quality of life (SF-12) and a reduction in analgesic intake for pain control. The study also observed a statistically significant difference in the percentage of body fat and muscle mass measured by bioelectrical impedance analysis [35].

The superior efficacy of triple HMW low dose injection over single injections can be explained by several factors. Firstly, the total amount of injected HA in the triple HMW low dose group was 90 mg, which could have contributed to its better performance compared to multiple injections. Additionally, this triple injection approach may have provided sustained release of HA into the joint, resulting in prolonged anti-inflammatory and chondroprotective effects. Previous studies have shown that repeated injections of HA can increase the concentration of HA in the synovial fluid, leading to longer-lasting effects on cartilage metabolism. Furthermore, the use of low-dose injections may have resulted in better distribution of HA within the joint, leading to more uniform effects on the cartilage and synovium. On the other hand, the single injection group containing a total of 60 mg of high molecular weight (HMW) HA also showed promising results, similar to multiple injections. The increasing use of HMW-containing HA and the preference for higher doses of HA have expanded the treatment options for doctors, allowing them to choose between single and multiple injection approaches based on the individual clinical findings of each patient.

The main limitation of the study is its retrospective design, which may introduce potential biases and limitations in data collection and analysis. However, a strength of the study is the prospective collection of data, which helps to enhance the reliability and accuracy of the information gathered. Another limitation of the study is the absence of a comparison group receiving a different molecule other than HA. To ensure homogeneity of the patient population in our study, patients with knee osteoarthritis between the ages of 50 and 60 were included. However, this represents a specific group within the patient population suffering from knee osteoarthritis and is a limitation of our study. The patient selection process for group formation introduces a methodological bias, constituting a limitation in our study. Having a control group with an alternative treatment or placebo would have allowed for a more robust comparison of the effectiveness of HA injections in knee osteoarthritis. It is essential to acknowledge these limitations as they may impact the generalizability and interpretation of the study findings.

## Conclusion

Our findings suggest that the triple low-dose injection of HMW HA is more effective in improving WOMAC, VAS, and Lequesne Index scores than a single high-dose injection. However, the high-dose HA group demonstrated faster pain reduction and an earlier effect. In addition, this study is one of the few studies in the literature in terms of using injections with high density and high HA content and having a clinical follow-up of at least 12 months. Therefore, this study may contribute to the literature on the efficacy of HA injections in OA patients, especially with long-term follow-up.

Both single and multiple intra-articular injections of HA are efficient treatments to reduce pain and improve function in knee osteoarthritic patients.

Efficacy is generally observed up to 6 months, then, as pain and handicap reappear before 1 year, it could be advisable to renew the treatment, at the patient's convenience.

## Data Availability

The datasets of current study are available from corresponding author on a reasonable request. All analysed study-related data are included in this article.

## Abbreviations

HA: Hyaluronic acid
HMW: High-molecular weight
NSAII: Non-steroidal anti-inflammatory
OA: Osteoarthritis
PRP: Platelet-rich plasma
VAS: Visual analog scale
WOMAC: Western Ontario and McMaster Universities arthritis index
